# Survival analysis of laparoscopic and laparotomy hepatectomy for pediatric liver neoplasm: a single-center retrospective cohort study

**DOI:** 10.3389/fped.2026.1892867

**Published:** 2026-07-23

**Authors:** Shan Huang, Qianwei Yang, Zhenyu Xie, Yang Chen, Yun Peng, Chengkun Luo, Jiulin Song, Jian Yang

**Affiliations:** 1 Department of Nephrology, Sichuan Provincial People’s Hospital, University of Electronic Science and Technology of China, Chengdu, China; 2 School of Clinical Medicine, The First Affiliated Hospital of Chengdu Medical College, Chengdu, China; 3 Department of Pediatric Surgery, West China Hospital, Sichuan University, Chengdu, China; 4 Department of Liver Transplantation Center, West China Hospital, Sichuan University, Chengdu, China

**Keywords:** benign liver neoplasm, hepatectomy, laparoscopic surgery, malignant liver neoplasm, pediatric liver neoplasm

## Abstract

**Objective:**

To evaluate the efficacy and postoperative survival following laparoscopic and laparotomy hepatectomy of pediatric liver neoplasm (PLN).

**Methods:**

A retrospective analysis was conducted on 180 patients who underwent hepatectomy of PLN in our center between January 2014 and May 2025. Demographic information, perioperative clinical data, postoperative liver function recovery, complications, and survival outcomes were collected to evaluate the efficacy, postoperative complications, and survival rates after laparoscopic and laparotomy hepatectomy of PLN.

**Results:**

In this study, 89 patients (49.44%) were male and 91 (50.56%) were female. The median age at surgery was 5.7 years. 66 patients (36.67%) were diagnosed with benign liver neoplasms (BLN), and 114 patients (63.33%) with malignant liver neoplasms (MLN). Laparoscopy was performed in 83 patients (46.11%), while laparotomy was performed in 97 patients (53.89%). In the BLN group, there were no significant differences between the laparoscopic and laparotomy groups in terms of postoperative liver function recovery or complication rates. The postoperative survival rate was 100% in both groups. However, the length of hospital stay was significantly shorter in the laparoscopic group (*P* < 0.001). In the MLN group, there were no significant differences in postoperative liver function recovery or complication rates between the two surgical approaches. However, the laparoscopic group had a significantly longer operative time (*P* = 0.009), more frequent Pringle maneuver (*P* < 0.001). The 5-year cumulative survival rate was 78.13% in the laparoscopic group and 81.23% in the laparotomy group, with no significant difference between the two approaches (*P* = 0.732).

**Conclusion:**

Surgical treatment for PLN yields favorable outcomes. Laparoscopy is recommended for BLN or MLN with localized lesions. However, for large MLN that encase vessels or show diffuse infiltration, laparotomy is recommended.

## Highlights

- Laparoscopy and laparotomy show comparable safety and survival in pediatric liver neoplasms.
- Laparoscopy reduces hospital stay in benign tumors.
- Laparotomy is preferred for large or vascular-involved malignant tumors.

## 1 Introduction

The incidence of pediatric liver neoplasms is relatively low (1, 2). Among malignant neoplasms, hepatoblastoma (HB) is the most common in children, followed by hepatocellular carcinoma (HCC) (3, 4). These two types account for approximately 80% of pediatric malignant liver neoplasms. Among benign neoplasms, the most common are hepatic hemangioma (HH) and focal nodular hyperplasia (FNH) (5). Hepatectomy remains the cornerstone of treatment for pediatric MLN, with neoadjuvant chemotherapy and chemotherapy serving as important adjunct therapies (6–9). For BLN with significant clinical symptoms, surgical intervention is also necessary (10). In this study, a retrospective analysis was performed on PLN hepatectomy conducted at our center. Clinical data were compared to evaluate the efficacy, postoperative complications, and survival rates after laparoscopic and laparotomy.

## 2 Data and methods

### 2.1 Clinical data

A retrospective analysis was conducted on patients who underwent laparoscopic or laparotomy hepatectomy for PLN at the Department of Pediatric Surgery, West China Hospital of Sichuan University, between January 2014 and May 2025.

Inclusion criteria were as follows: (1) Diagnosis of a liver neoplasm based on clinical history, symptoms, physical examination, abdominal Doppler ultrasound, contrast-enhanced abdominal CT or MRI, and confirmed by postoperative pathology; (2) Absence of diffuse intrahepatic disease and severe dysfunction of extrahepatic organs, with sufficient condition to tolerate general anesthesia; (3) Preoperative evaluation by a multidisciplinary team confirming suitability for laparoscopic or laparotomy hepatectomy; (4) Informed consent for surgery signed by the patient's parents before the operation. Exclusion criteria included: (1) Patients with advanced PLN with poor general condition; (2) Incomplete clinical data.

Clinical data were collected for each patient, including demographic information: sex, age, and weight; perioperative variables: total bilirubin(TB), direct bilirubin(DB), alanine aminotransferase(ALT), aspartate aminotransferase(AST), albumin(ALB), prothrombin time(PT), surgical approach, operative time(OT), intraoperative blood loss(BL), intraoperative transfusion volume(ITV), duration of Pringle maneuver(PM), type of hepatectomy, and length of hospital stay(HS); liver function recovery at one week postoperatively; and postoperative complications, such as hypoproteinemia(HP), intra-abdominal bleeding(IAB), intra-abdominal infection(IAI), adhesive intestinal obstruction(AIO), and bile leakage(BL). In response to surgical selection considerations, additional tumor-related baseline characteristics were also collected, including maximum tumor diameter, tumor location, and, for malignant liver neoplasms, PRETEXT stage and major vascular involvement when available. Tumor size was defined as the maximum diameter measured on preoperative imaging. Tumor location and vascular involvement were assessed based on contrast-enhanced CT, MRI, and operative records. Major vascular involvement included involvement or encasement of the portal vein, hepatic veins, inferior vena cava, or hepatic artery. Surgical margin status was reviewed from postoperative pathological reports and categorized as R0 resection or R1 resection. R0 resection was defined as microscopically negative surgical margins, whereas R1 resection was defined as microscopically positive margins. Patient prognosis was evaluated through postoperative follow-up.

This study was approved by the Ethics Committee of West China Hospital, Sichuan University (Approval No. 2024-11). Informed consent was obtained from the legal guardians of all patients.

### 2.2 Case grouping

Based on auxiliary examinations and pathological outcomes, patients were classified according to the WHO classification of PLN. They were divided into BLN group (including HH, FNH, and mesenchymal hamartoma (MH)) and MLN group (including HB, HCC, undifferentiated embryonal sarcoma (UES), and inflammatory myofibroblastic tumor (IMT)).

Perioperative data, liver function recovery at one week after surgery, postoperative complications, and survival rates were compared between patients in the BLN and MLN groups who underwent either laparoscopy or laparotomy.

### 2.3 Preoperative management

All patients underwent standard preoperative evaluations, including complete blood count, liver and renal function tests, electrolytes, coagulation profile, full pre-transfusion screening, tumor markers, and at least one hepatobiliary imaging examination. For MLN, primarily HB, hepatectomy was considered in patients with PRETEXT stage I, II, and selected stage III tumors. Resection was indicated when a negative surgical margin of more than 1 cm and an expected remnant liver volume greater than 25% of standard liver volume (or >30% after chemotherapy) could be achieved. Neoadjuvant chemotherapy was routinely administered before surgery and continued postoperatively in MLN cases. For BLN, surgical indications included the presence of clinical symptoms, risk of rupture, or inability to rule out malignancy. Contraindications for hepatectomy included: (1) MLN involving major vascular structures (such as the portal vein, hepatic veins, or inferior vena cava), presence of tumor thrombus, extrahepatic metastasis, or insufficient future liver remnant; (2) Poor general condition of patients, making them unable to tolerate general anesthesia. The choice between laparoscopic or laparotomy was made by the guardians of patients based on informed decision-making.

For patients with malignant liver neoplasms, neoadjuvant and postoperative chemotherapy were administered according to tumor type, disease stage, and multidisciplinary team recommendations. In this cohort, 32/114 patients (28.07%) with malignant liver neoplasms received preoperative chemotherapy. Postoperative chemotherapy was continued when indicated based on pathological diagnosis, surgical margin status, tumor response, and oncological assessment.

### 2.4 Surgical approaches

For laparotomy, a right subcostal incision or a transverse subcostal incision (for large LN) was made. After opening the abdomen, the surrounding hepatic ligaments were dissected. The extent of hepatectomy was determined based on intraoperative exploration. Foley catheter was placed to constrict hilar structures. For patients undergoing localized hepatectomy (such as segmental or partial LN resection), hepatic inflow occlusion was not routinely performed. Liver parenchyma was transected layer by layer along the resection line using an ultrasonic scalpel. Hemostasis of the liver transection surface was achieved with either a microwave scalpel or saline-linked bipolar cautery. Intrahepatic vessels with a diameter less than 2 mm were coagulated and divided directly. Vessels larger than 2 mm were ligated with silk sutures before division.

For laparoscopic surgery, the patient was placed in a 30° left lateral decubitus position with a head-up and feet-down tilt. 5-mm laparoscopes was used, and the “five-port technique” was commonly applied. Pneumoperitoneum pressure was maintained below 12 mmHg. Foley catheter was placed to constrict hilar structures. For hemihepatectomy, the left or right hepatic artery, portal vein, and hepatic duct were dissected within the extrahepatic Glisson's sheath. These structures were ligated with silk sutures or clipped with Hem-o-Lok clips before division. The liver parenchyma was then transected along the demarcation line of hepatic ischemia to remove the neoplasm. For non-anatomical hepatectomy, Foley catheter was placed. Intraoperative ultrasound was used to locate the neoplasm and define the resection margin. The neoplasm was resected with a 0.5∼1 cm margin from its edge. Intrahepatic Vessels smaller than 2 mm in diameter were divided directly by ultrasonic scalpel. Vessels larger than 2 mm were clipped with Hem-o-Lok clips before division. After neoplasm resection, the specimen was placed in a plastic bag. Specimens were removed intact through an enlarged trocar site or a Pfannenstiel incision. BLN were morcellated and removed piecemeal through a 12-mm trocar port.

The Pringle maneuver was not routinely applied in all patients. In our institution, it was selectively used when tumor location, expected transection plane, proximity to major vascular structures, or intraoperative bleeding risk suggested the need for temporary hepatic inflow control. The decision to apply the Pringle maneuver, as well as the number and duration of inflow occlusions, was made intraoperatively by the operating surgeon according to case complexity, bleeding risk, and surgical field exposure.

### 2.5 Statistical analysis

Statistical analysis was performed using SPSS version 27.0 and GraphPad Prism version 10.1.2. The normality of quantitative data was assessed. Data with a normal distribution were expressed as mean ± standard deviation ($\bar{X}$±SD), and intergroup comparisons were conducted using the independent-samples t-test. Non-normally distributed data were expressed as median with interquartile range M (Q1, Q3), and the Mann–Whitney U test was used for comparisons between groups. Qualitative data were expressed as frequencies. The Pearson chi-square test was used for comparisons between groups. If the assumptions for the Pearson test were not met, the continuity-corrected chi-square test was applied. *P* < 0.05 was considered statistically significant. Kaplan–Meier survival curves were used to perform Log-Rank survival analysis between laparoscopic and laparotomy groups in both BLN and MLN cohorts.

## 3 Results

### 3.1 Clinical characteristics of 180 patients undergoing hepatectomy

Among the 180 patients included in this study, 89 were male (49.44%) and 91 were female (50.56%). The median age was 5.7 (2.5∼10.8) years, and the median weight was 18.5(11.9–33.9) kg. Regarding the primary diagnosis, 114 patients (63.33%) was MLN. These included 77 cases of HB (42.8%), 34 cases of HCC (18.8%), 2 cases of UES (1.2%), and 1 case of IMT (0.6%). 66 patients (36.67%) were diagnosed with BLN, including 45 cases of FNH (25.0%), 12 cases of HH (6.6%), and 9 cases of MH (5.0%). Laparoscopic surgery was performed in 83 cases (46.11%), while traditional laparotomy was performed in 97 cases (53.89%). Anatomical hepatectomy was performed in 77 patients (42.78%), and non-anatomical (wedge or partial) hepatectomy was performed in 103 patients (57.22%). The BLN median maximum tumor diameter was 9.2 cm. Regarding tumor location, 21(31.82%) tumors were located in the left liver, 37(56.06%) in the right liver, and 8(12.12.%) showed bilobar involvement. Among patients with malignant liver neoplasms, median maximum tumor diameter was 8.6 cm. Regarding tumor location, 22(19.30%) tumors were located in the left liver, 78(68.42%) in the right liver, and 14(12.28%) showed bilobar involvement. The distribution of PRETEXT stages was as follows: PRETEXT I in 62(54.39%) patients, PRETEXT II in 41(35.96%) patients, and PRETEXT III in 11(9.65%) patients. Major vascular involvement was identified in 23(19.83%) patients (Table 1).

**Table 1:** Clinical characteristics of 180 patients undergoing hepatectomy.

Baseline data	Characteristics
Age (y)	5.7 (2.5,10.8)
Gender *(n, %)*	
Male	89 (49.44%)
Female	91 (50.56%)
Weight (kg)	18.5 (11.9,33.9)
Diagnosis *(n, %)*	
HB	77 (42.8%)
HCC	34 (18.8%)
UES	2 (1.2%)
IMT	1 (0.6%)
HH	12 (6.6%)
FNH	45 (25%)
MH	9 (5%)
Surgical approaches	
Laparoscopy	83 (46.11%)
Laparotomy	97 (53.89%)
Hepatectomy types	
Anatomical hepatectomy	77 (42.78%)
Non-anatomical hepatectomy	103 (57.22%)
Malignant tumor characteristics	
Tumor size	8.6 (4.3, 13.5) cm
Tumor location	
Left liver	22 (19.30%)
Right liver	78 (68.42%)
Bilobar involvement	14 (12.28%)
PRETEXT stage	
PRETEXT I	62 (54.39%)
PRETEXT II	41 (35.96%)
PRETEXT III	11 (9.65%)
PRETEXT IV	0
Vascular involvement	23 (19.83%)
Benign tumor characteristics	
Tumor size	9.2 (6.3, 15.6) cm
Tumor location	
Left liver	21 (31.82%)
Right liver	37 (56.06%)
Bilobar involvement	8 (12.12.%)

### 3.2 Comparative analysis of clinical data between laparoscopic and laparotomy groups with BLN

Among the 66 patients with BLN, 38 underwent laparoscopic surgery and 28 underwent traditional laparotomy. As shown in Table 2, there were no statistically significant differences between the laparoscopic and laparotomy groups in terms of Gender (*P* = 0.681), age (*P* = 0.134), weight (*P* = 0.054), preoperative of TB (*P* = 0.132), DB (*P* = 0.640), ALT (*P* = 0.056), AST (*P* = 0.086), ALB (*P* = 0.175), PT (*P* = 0.222), OT (*P* = 0.425), IBL (*P* = 0.613), ITV (*P* = 0.109), times of PM (*P* = 0.066), duration of PM (*P* = 0.101), or type of hepatectomy (*P* = 0.356). However, the mean length of HS was significantly shorter in the laparoscopic group (7.3 ± 1.2d) compared to the laparotomy group (10.6 ± 2.1d), with a statistically significant difference (*P* < 0.001).

**Table 2:** Comparison of clinical data between laparoscopic and laparotomy groups with BLN.

Clinical data	Laparoscopy (*n* = 38)	Laparotomy (*n* = 28)	*P*	Test statistic
Gender (*n*)			0.618	0.248
Male	20	13		
Female	18	15		
Age (y)	7.1 (5.2,12.1)	6.9 (5.1,11.9)	0.134	−1.499
Weight (kg)	24.1 (18.9,27.5)	23.5 (18.5,26.8)	0.054	−1.927
TB (μmol/L)	15.5 (10.3,21.2)	14.8 (10.1,20.9)	0.132	−1.505
DB (μmol/L)	4.8 (2.1,7.5)	5.1 (2.2,8.1)	0.640	−0.467
ALT (U/L)	28 (14,49)	25 (13,47)	0.056	−1.908
AST (U/L)	31 (16,55)	30 (14,54)	0.086	−1.720
ALB (g/L)	41 (35,49)	40 (35,48)	0.175	−1.357
PT (s)	12.2 (11.2,13.9)	12.5 (11.1,13.8)	0.222	−1.220
OT (min)	125 (92,165)	137 (89,175)	0.425	−0.798
IBL (mL)	55 (32,71)	59 (33,75)	0.613	−0.506
ITV (U)	0.8 ± 0.2	0.9 ± 0.3	0.109	1.624
PM (times)	2.8 ± 0.5	3.1 ± 0.8	0.066	1.871
PM (min)	42.2 ± 7.1	45.6 ± 9.5	0.101	1.665
Hepatectomy types (*n*)			0.356	0.851
Anatomy	16	15		
Non-anatomy	22	13		
HS (d)	7.3 ± 1.2	10.6 ± 2.1	<0.001	8.074
Liver function recovery one week after surgery				
TB (μmol/L)	12.1 ± 4.6	11.7 ± 4.5	0.726	0.352
DB (μmol/L)	3.3 ± 1.4	3.5 ± 1.5	0.580	0.557
ALT (U/L)	20.2 ± 8.1	21.3 ± 7.9	0.583	0.551
AST (U/L)	25.5 ± 8.9	24.9 ± 9.1	0.789	0.268
ALB (g/L)	42.2 ± 3.7	41.8 ± 3.2	0.648	0.459
PT (s)	12.1 ± 0.8	12.3 ± 0.7	0.294	1.057
Postoperative complications				
HP (*n*)	3	2	0.909	0.013
IAI (*n*)	2	1	0.744	0.106
AIO (*n*)	0	2	0.176	2.799

Liver function returned to normal levels in both groups one week after surgery. As shown in Table 2 (Liver Function Recovery after A Week of Postoperative), there were no statistically significant differences between the laparoscopic and laparotomy groups in the postoperative recovery of TB (*P* = 0.726), DB (*P* = 0.580), ALT (*P* = 0.583), AST (*P* = 0.789), ALB (*P* = 0.648), or PT (*P* = 0.294).

Regarding postoperative complications, 3 patients in the laparoscopic group developed HP (7.89%), and 2 developed IAI (5.26%). In the laparotomy group, 2 patients had HP (7.14%), 1 had IAI (3.57%), and 2 developed AIO (7.14%). There were no statistically significant differences between the two groups in the incidence of HP (*P* = 0.909), IAI (*P* = 0.744), or AIO (*P* = 0.176) (Table 2 Postoperative Complications).

**Figure 1:**
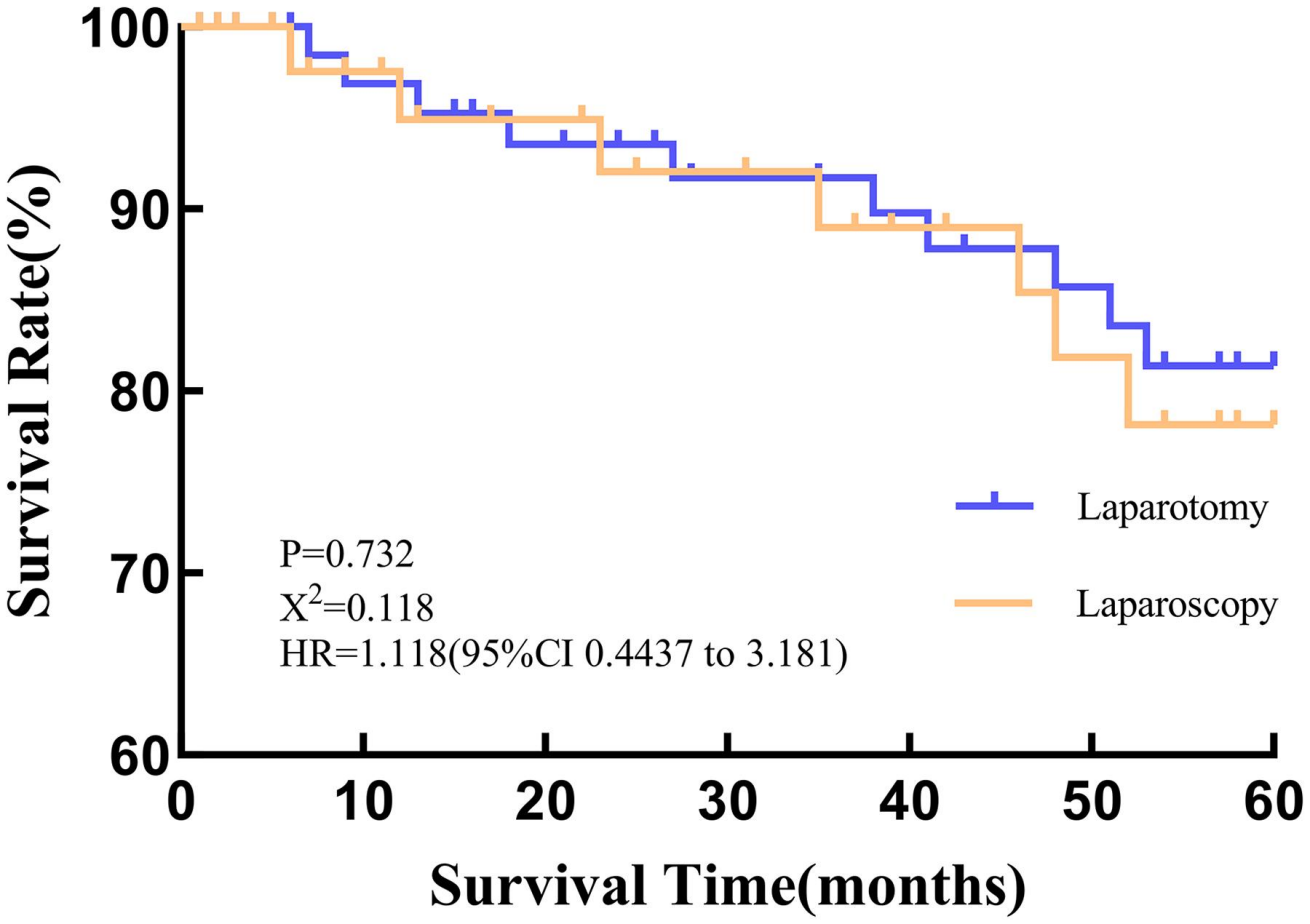
Kaplan–Meier survival analysis after laparoscopic and laparotomy operation with BLN.

In terms of postoperative survival, no deaths occurred in either group of patients with BLN who underwent laparoscopic or laparotomy. The survival rate was 100% in both groups (Figure 1).

### 3.3 Comparative analysis of clinical data between laparoscopic and laparotomy groups with MLN

Among the 114 patients with MLN, 45 underwent laparoscopic surgery and 69 underwent traditional laparotomy. As shown in Table 3, there were no statistically significant differences between the laparoscopic and laparotomy groups in terms of gender (*P* = 0.420), age (*P* = 0.407), weight (*P* = 0.329), preoperative of TB (*P* = 0.256), DB (*P* = 0.600), ALT (*P* = 0.131), AST (*P* = 0.102), ALB (*P* = 0.478), PT (*P* = 0.069), IBL (*P* = 0.702), ITV (*P* = 0.154), type of hepatectomy (*P* = 0.951), or length of HS (*P* = 0.174). However, the median operative time was significantly longer in the laparoscopic group 145(103∼185) minutes compared to the laparotomy group 128(95∼169) minutes (*P* = 0.009). The mean times of PM was 4.9 ± 0.7 in the laparoscopic group and 3.9 ± 0.6 in the laparotomy group. The mean duration of PM was 70.1 ± 10.5 min and 58.9 ± 9.8 min, respectively. Both the times of PM (*P* < 0.001) and PM duration (*P* < 0.001) were significantly higher in the laparoscopic group than in the laparotomy group.

**Table 3:** Comparison of clinical data between laparoscopic and laparotomy groups with MLN.

Clinical data	Laparoscopy (*n* = 45)	Laparotomy (*n* = 69)	*P*	Test statistic
Gender (*n*)			0.420	0.651
Male	20	36		
Female	25	33		
Age (y)	5.1 (3.5,9.6)	4.7 (3.1,9.8)	0.407	−0.829
Weight (kg)	18.8 (14.1,25.5)	17.5 (12.1,24.9)	0.329	−0.977
TB (μmol/L)	18.9 (13.3,29.2)	19.1 (12.9,28.9)	0.256	−1.136
DB (μmol/L)	6.9 (4.1,10.6)	7.1 (4.4,12.1)	0.600	−0.525
ALT (U/L)	38 (29,85)	39 (27,96)	0.131	−1.512
AST (U/L)	35 (21,76)	34 (19,74)	0.102	−1.636
ALB (g/L)	36 (32,41)	35 (31,42)	0.478	−0.710
PT (s)	12.9 (11.5,14.4)	13.1 (11.9,14.9)	0.069	−1.815
OT (min)	145 (103,185)	128 (95,169)	0.009	−2.597
IBL (mL)	62 (39,80)	65 (41,84)	0.702	−0.386
ITV (U)	1.1 ± 0.3	1.2 ± 0.4	0.154	1.143
PM (times)	4.9 ± 0.7	3.9 ± 0.6	<0.001	8.140
PM (min)	70.1 ± 10.5	58.9 ± 9.8	<0.001	5.798
Hepatectomy types (*n*)			0.951	0.004
Anatomy	18	28		
Non-anatomy	27	41		
HS (d)	13.9 ± 2.8	14.7 ± 3.2	0.174	1.369
Liver function recovery after a week of postoperative				
TB (μmol/L)	10.8 (4.9,19.9)	11.2 (5.5,18.8)	0.717	−0.371
DB (μmol/L)	5.1 (4.2,8.1)	5.3 (4.1,7.9)	0.485	0.701
ALT (U/L)	18 (11,38)	19 (10,41)	0.975	0.035
AST (U/L)	15 (9,39)	14 (9,35)	0.612	0.510
ALB (g/L)	39 (35,43)	38 (35,44)	0.586	0.048
PT (s)	11.5 (10.9,13.4)	11.9 (10.7,13.6)	0.830	−0.217
Postoperative complications				
HP (*n*)	6	8	0.782	0.076
IAI (*n*)	4	6	0.972	0.001
IAB (*n*)	3	4	0.850	0.036
AIO (*n*)	5	7	0.869	0.027
BL (*n*)	2	2	0.661	0.192

Liver function in both groups had generally returned to normal levels one week after surgery. There were no statistically significant differences between the laparoscopic and laparotomy groups in the postoperative recovery of TB (*P* = 0.717), DB (*P* = 0.485), ALT (*P* = 0.975), AST (*P* = 0.612), ALB (*P* = 0.586), or PT (*P* = 0.830) (Table 3 Liver Function Recovery after A Week of Postoperative).

Regarding postoperative complications, 6 patients in the laparoscopic group developed HP (13.33%), 4 developed IAI (8.89%), 3 experienced IAB (6.67%), 5 developed AIO (11.11%), and 2 experienced BL (4.44%). In the laparotomy group, 8 patients developed HP (11.59%), 6 had IAI (8.70%), 4 experienced IAB (5.80%), 7 developed AIO (10.14%), and 2 experienced BL (2.90%). There were no statistically significant differences between the two groups in the incidence of HP (*P* = 0.782), IAI (*P* = 0.972), IAB (*P* = 0.850), AIO (*P* = 0.869), or BL (*P* = 0.661) (Table 3 Postoperative Complications).

Regarding postoperative MLN recurrence, metastasis, and mortality, 8 patients in the laparoscopic group experienced recurrence and metastasis (17.78%), while 13 patients in the laparotomy group had recurrence (18.84%). There were 7 deaths in the laparoscopic group (15.56%) and 10 deaths in the laparotomy group (14.49%). There were no statistically significant differences between the two groups in postoperative MLN recurrence and metastasis (*P* = 0.886) or mortality (*P* = 0.876). Among patients with malignant liver neoplasms, R0 resection was achieved in 40/45 patients (88.89%) in the laparoscopic group and 62/69 patients (89.86%) in the laparotomy group. R1 resection was observed in 5 and 7 patients, respectively. There was no statistically significant difference in R0 resection rate between the two groups (*P* = 0.831) (Table 4).

**Table 4:** Comparison of margin status, MLN recurrence, metastasis, and survival after laparoscopic and laparotomy surgery.

Clinical data	Laparoscopy (*n* = 45)	Laparotomy (*n* = 69)	*P*	Test statistic
Recurrence or metastasis (*n*)	8	13	0.886	0.020
Mortality (*n*)	7	10	0.876	0.024
Margin status			0.831	0.046
R0	40	62		
R1	5	7		

**Figure 2:**
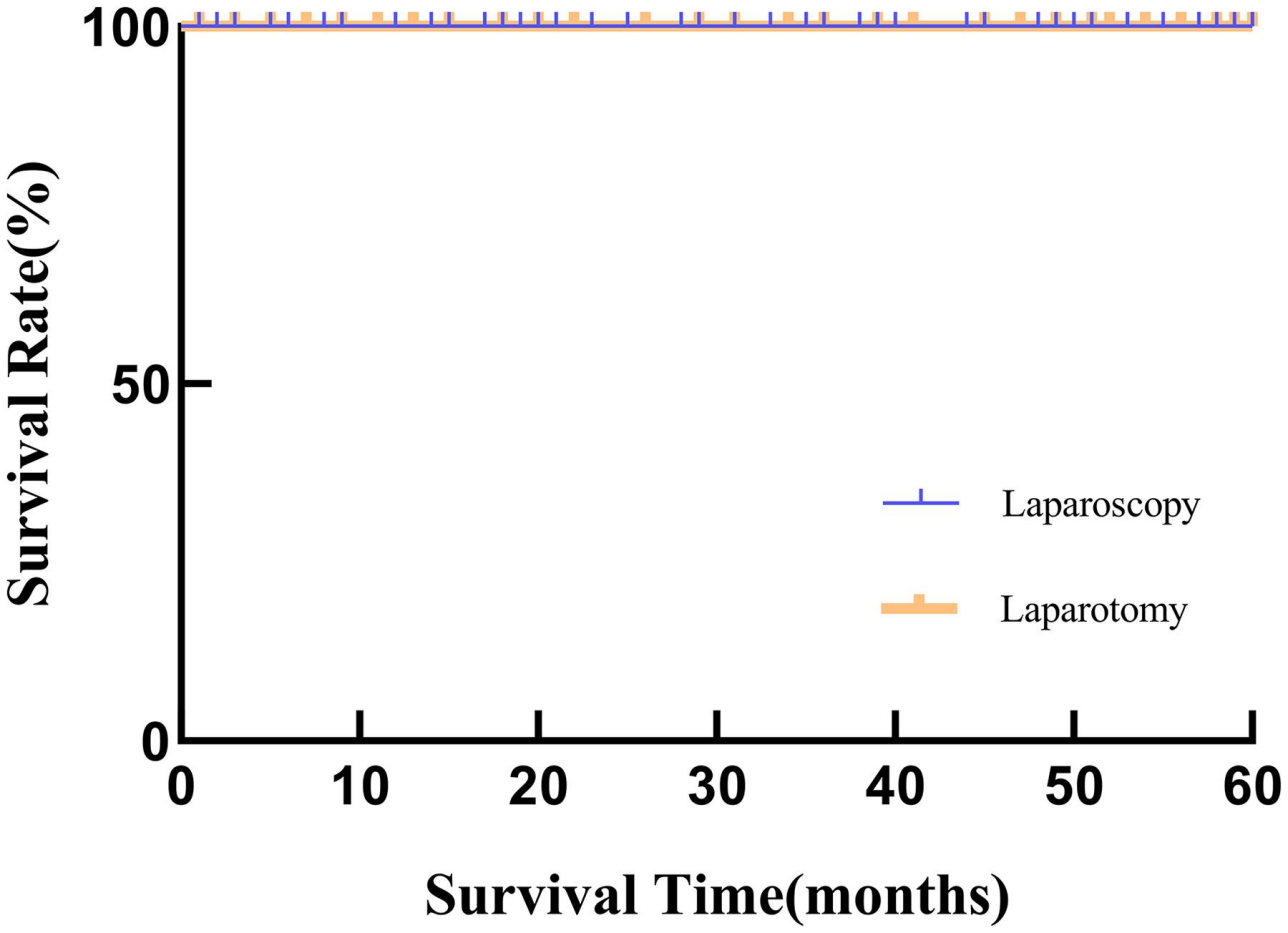
Kaplan–Meier survival analysis after laparoscopic and laparotomy operation with MLN.

In terms of postoperative survival, the median follow-up duration was 46 months, with a follow-up completeness rate of 95.5% for patients with BLN, and no deaths were observed during follow-up. For patients with MLN, the median follow-up duration was 48 months. At the final follow-up, recurrence or metastasis occurred in 8 patients in the laparoscopic group and 13 patients in the laparotomy group, while 7 and 10 deaths were observed in the two groups, respectively. The 1-, 3-, and 5-year cumulative survival rates in the laparoscopic group were 94.81%, 88.71%, and 78.13%. In the laparotomy group, the corresponding rates were 96.63%, 91.53%, and 81.23%. There was no statistically significant difference in 5-year survival between the two groups (*P* = 0.732) (Figure 2).

## 4 Discussion

Primary PLN are diverse and highly heterogeneous, which presents considerable challenges for surgical treatment. MLN account for approximately 60% of pediatric patient, with common types including HB, HCC, malignant mesenchymal tumors, and rhabdomyosarcoma (11–14). BLN comprise about 40%, and the major types include FNH, HH, mesenchymal hamartoma, and hepatic adenoma (3, 15–17). In addition, PLN often exhibit rapid growth, relatively large neoplasm size, complex anatomical location, and limited intra-abdominal operative space. These factors significantly increase the technical difficulty of surgical resection and limit the widespread application of laparoscopic techniques in hepatectomy.

In 1991, Reich et al (18) first reported laparoscopic resection of a BLN. Since then, with the advancement of minimally invasive surgical techniques and the continuous improvement of laparoscopic instruments, laparoscopic hepatectomy has been widely adopted for the resection of both BLN and MLN in adults and has been recommended by clinical guidelines (19, 20). However, due to the relatively low incidence of PLN, limited intra-abdominal space in children, greater technical difficulty, and a longer learning curve, the application of laparoscopic techniques in PLN hepatectomy has progressed more slowly. Only a few centers perform these procedures routinely, and the overall number of cases remains limited (21). In the early 2000s, pediatric laparoscopic surgery was mainly used for liver biopsy (22, 23). With the development of minimally invasive hepatobiliary surgery and the improvement of laparoscopic instruments, the application of laparoscopic hepatectomy in PLN has gradually been reported. In 2006, Yoon et al (24) successfully performed laparoscopic left lateral hepatectomy in a 4-year-old girl with a ciliated foregut cyst in the left lateral segment of the liver. This case represented the first reported laparoscopic hepatectomy of a BLN in a child worldwide. In 2011, Kim et al (25) were the first to report two cases of MLN (HB) treated with laparoscopic hepatectomy, both with favorable outcomes. In recent years, our center has actively developed laparoscopic techniques for PLN hepatectomy. In 2020, our team (26) reported a series of 21 cases, demonstrating that laparoscopic hepatectomy can promote faster postoperative recovery, reduce pain, and does not increase the risk of postoperative complications.

In this study, 83 patients who underwent laparoscopic surgery successfully completed total laparoscopic hepatectomy of PLN. For BLN, the length of HS was significantly shorter in the laparoscopic group compared to the laparotomy group, which may be attributed to the enhanced recovery benefits associated with minimally invasive surgery. The concept of Enhanced Recovery After Surgery (ERAS) was first introduced by Henrik Kehlet (27) in 1997, aiming to accelerate the return to physiological function by optimizing perioperative management. Previous studies have shown (28–31) that postoperative organ dysfunction and complications are associated with surgical stress responses. Minimally invasive techniques, such as laparoscopy, help reduce immune dysfunction and surgical stress caused by operative trauma. BLN are typically expansile and exhibit minimal invasion or adhesion to surrounding tissues (15), which makes them relatively easier to resect laparoscopically. In this study, no significant differences were found between the laparoscopic and laparotomy groups in terms of postoperative liver function recovery or complication rates. Additionally, laparoscopic surgery offers the benefit of improved cosmetic outcomes. Therefore, when performed by experienced surgeons with proficiency in both liver surgery and laparoscopic techniques, laparoscopic hepatectomy should be considered the preferred approach for BLN, as it may provide greater clinical benefit.

In cases of MLN, this study found that OT was significantly longer in the laparoscopic group compared to the laparotomy group. Additionally, both the times and duration of PM were greater in the laparoscopic group. These findings are related to the biological behavior of MLN and the complexity of hepatectomy. MLN often exhibit infiltrative growth patterns and are tightly adherent to surrounding tissues (32), which increases the difficulty of tissue dissection during laparoscopic surgery and contributes to longer OT. These MLN also frequently encase major vascular structures, such as the hepatic artery, hepatic veins, and the portal venous system, as well as the biliary system (33). Therefore, in laparoscopic hepatectomy of MLN, PM is used more frequently and for longer durations to control intraoperative bleeding. Moreover, due to the smaller abdominal cavity and more fragile organs in children, laparoscopic procedures require more delicate and meticulous manipulation to minimize trauma. As a result, OT tend to be longer, and the technical demands on the surgeon are higher. Despite these challenges, there were no statistically significant differences between the laparoscopic and laparotomy groups in terms of postoperative liver function recovery or complication rates. This supports the safety and feasibility of laparoscopic surgery for pediatric MLN. With respect to MLN outcomes, the 1-, 3-, and 5-year cumulative survival rates in the laparoscopic group were 94.81%, 88.71%, and 78.13%, respectively. In the laparotomy group, the corresponding rates were 96.63%, 91.53%, and 81.23%. A previous study (34) reported a 5-year survival rate of approximately 60% following complete resection of MLN, although that study was conducted many years ago. More recent studies (11, 35–40) have shown 5-year survival rates ranging from 70% to 90% in pediatric patient, which is consistent with the outcomes reported by our center. These results suggest that advances in surgical techniques have contributed to substantial improvements in long-term survival following complete resection of MLN. Although the laparoscopic group had longer OT and greater use of PM, these differences are expected to diminish with increasing surgical experience and technical refinement.

The more frequent and longer use of the Pringle maneuver in the laparoscopic group should be interpreted with caution. In our center, the Pringle maneuver was applied selectively rather than routinely. Its use was influenced by tumor location, the complexity of liver parenchymal transection, proximity to major vascular structures, intraoperative bleeding risk, and surgeon judgment. Therefore, the greater use of the Pringle maneuver in the laparoscopic group may reflect both the technical demands of laparoscopic liver transection and case-specific complexity rather than the surgical approach alone. Although no significant differences in postoperative liver function recovery or complication rates were observed between the two groups, the potential influence of surgeon preference and intraoperative decision-making should be considered when interpreting these findings.

Laparoscopic hepatectomy in pediatric patient is technically demanding and carries a higher surgical risk. Intraoperative prevention of massive bleeding and accurate localization of PLN margins are among the most critical challenges in pediatric laparoscopic hepatectomy (41). Strict preoperative patient selection is essential. Laparoscopic hepatectomy is most appropriate for BLN or MLN that are well localized (42, 43). Based on our center's experience, intraoperative techniques such as PM to constrict hilar structures, controlling central venous pressure below 5 cmH₂O, or maintaining pneumoperitoneum pressure above 9 mmHg can effectively reduce intraoperative bleeding.

Several limitations should be acknowledged. First, this was a retrospective single-center study, and the choice of laparoscopic or Laparotomy hepatectomy was not randomized. Although all patients underwent preoperative multidisciplinary evaluation, the final surgical approach was influenced by tumor size, tumor location, PRETEXT stage, vascular involvement, surgical feasibility, surgeon experience, and parental preference. Therefore, selection bias could not be completely avoided. Patients selected for laparoscopic hepatectomy may have had more favorable tumor characteristics, whereas patients with larger tumors, diffuse infiltration, or major vascular involvement were more likely to undergo Laparotomy. Second, although we supplemented tumor-related baseline characteristics and surgical margin status, the retrospective design still limited the standardization of imaging assessment, chemotherapy protocols, and surgical selection criteria. Therefore, although recurrence, metastasis, mortality, and long-term survival were compared between the two groups, the oncological adequacy of resection could not be fully evaluated in all cases. Third, the cohort included heterogeneous pathological subtypes of pediatric liver neoplasms, and subgroup analyses were limited by the rarity of these tumors. Future prospective multicenter studies with standardized surgical selection criteria, complete oncological variables, and risk-adjusted analyses are needed to further validate these findings.

## 5 Conclusion

Surgical treatment of pediatric liver neoplasms generally yields favorable outcomes. In selected patients with benign liver neoplasms or localized malignant liver neoplasms, laparoscopic hepatectomy appears to be a safe and feasible approach when performed by experienced surgeons. It may provide the benefits of minimally invasive surgery, including shorter hospital stay and improved cosmetic outcomes. However, for large malignant liver neoplasms, tumors with major vascular encasement, or lesions with diffuse infiltration, laparotomy remains a more appropriate surgical approach. Because of the retrospective design and non-randomized surgical selection, these findings should be interpreted with caution and further validated in prospective multicenter studies.

## Data Availability

The raw data supporting the conclusions of this article will be made available by the authors, without undue reservation.
